# Experiencing (Shared) Decision Making: Results from a Qualitative Study of People with Mental Illness and Their Family Members

**DOI:** 10.3390/healthcare11162237

**Published:** 2023-08-09

**Authors:** Katja Schladitz, Elena C. Weitzel, Margrit Löbner, Bettina Soltmann, Frank Jessen, Andrea Pfennig, Steffi G. Riedel-Heller, Uta Gühne

**Affiliations:** 1 Institute of Social Medicine, Occupational Health and Public Health (ISAP), Medical Faculty, Leipzig University, 04103 Leipzig, Germany; elena.weitzel@medizin.uni-leipzig.de (E.C.W.); margrit.loebner@medizin.uni-leipzig.de (M.L.); steffi.riedel-heller@medizin.uni-leipzig.de (S.G.R.-H.); uta.guehne@medizin.uni-leipzig.de (U.G.); 2 Department of Psychiatry and Psychotherapy, Medizinische Fakultät Carl Gustav Carus, TU Dresden, 01307 Dresden, Germany; 3 Department of Psychiatry and Psychotherapy, Faculty of Medicine and University Hospital Cologne, University of Cologne, 50937 Köln, Germany

**Keywords:** shared decision making, empowerment, trialogical approach, mental illness, mental health

## Abstract

(1) Background: There is a fundamental shift in healthcare toward shared decision making (SDM). This study explores SDM from the perspective of individuals affected by mental illness and their family members and investigates factors which promote and hinder the process. (2) Methods: We conducted *N* = 15 telephone interviews (*n* = 4 adults affected by mental illness, *n* = 5 family members, *n* = 6 both applicable, the majority reporting experiences with affective and anxiety disorders). Data were recorded, transcribed, and analyzed according to procedures established by Mayring. (3) Results: Individuals affected by mental illness and their family members have a strong desire to be involved in treatment decisions and to participate in finding a diagnosis. Often these stakeholders are denied the opportunity to participate; sometimes enabling behaviors impede participation. The stigmatization of mental illnesses is a major barrier. There are also structural barriers to SDM within the healthcare system. Peer support, self-help associations, and psychosocial counseling services are important to empowering individuals and promoting SDM. (4) Conclusions: SDM has the potential to improve the quality of mental healthcare. Barriers can be mitigated and new approaches for interventions in the psychiatric sector have been identified. This study has also shown the importance of understanding SDM as a process that should begin at the diagnostic phase.

## 1. Introduction

A fundamental change has begun in the mental healthcare sector in recent decades; the relationship between those affected by an illness and those treating them has been re-defined [1]. As affected individuals are increasingly seen as active, autonomous, and equal partners in all sectors of healthcare, participation has become a guiding principle in all aspects of healthcare, and the empowerment of patients has gained great importance [2,3].

There has been a shift from paternalistic (medical professionals deciding for patients) to shared (involving patients or clients as well as family members) decision-making (SDM) processes in medical encounters, including in mental healthcare [4,5,6,7,8]. As first outlined by Charles et al. [9], SDM is a triadic form of collaboration between healthcare providers, who share empirical evidence, individuals affected by mental illness, and their family members. The aim of SDM is for providers to enable and empower patients and families to make informed decisions that suit their unique personal and social situation [10]. Charles et al. [9] described the characteristics of SDM as follows: All involved parties share information, discuss benefits and potential harms of treatment options, express individual preferences, build a consensus, and agree to implement treatment. Gurtner et al. [4] emphasized that SDM refers to a patient-centered process which is usually not limited to a single consultation and that includes all relevant types of healthcare workers (including peers), as well as family members and friends. SDM is a key element in patient- or user-centered care which focuses on the values and preferences of the individual affected by an illness who should be actively and indispensably engaged in healthcare decisions [11]. SDM is also an essential element in the recovery process because it promotes autonomy and self-determination [6,12]. Individuals affected by mental health problems indicate that they feel empowered by the recovery movement and increasingly demand to be involved in decisions concerning their treatment [13].

Individuals affected by mental illness perceive a positive effect on their psychological well-being when they join a shared-decision trialogue concerning their treatment [14]. There is positive empirical evidence for implementing SDM, such as increased compliance with treatment for depression [15], better psychiatric medication management [16,17], cost reductions resulting from decreased hospital admissions [17,18], and more efficient prescription of medications [19] for people with serious mental illness. There is evidence that engaging family members in SDM has positive effects on mental health outcomes such as improved compliance to treatment, fewer relapses in schizophrenia, and reduced hospital admissions [20,21,22]. Therefore, there is an increasing scientific consensus that families should be included in therapeutic decisions [23,24,25].

Despite these findings, research indicates that there are barriers to implementing the SDM approach in mental healthcare. Users of healthcare services report that they feel that they are not perceived as equal partners in the decision-making process [26] and many individuals with mental illness report unmet needs related to information and SDM [27]. Family members of affected individuals also often feel insufficiently involved in the treatment process at an equal level [28,29]. Barriers to SDM are found at all levels of care: individual (e.g., lack of confidence, insufficient risk assessment, or lack of trust in healthcare professionals on the side of individuals with mental illness and family members), structural (e.g., time constraints in medical consultations), and institutional (e.g., inadequate professional training of health providers for SDM) [30,31,32]. Most of these barriers are modifiable [32], e.g., on the individual side, by increasing awareness and knowledge about the impaired mental health of affected individuals and their family members [31].

There is a need for research exploring the experiences and needs of individuals affected by mental illness and family members regarding decision making. This is an important prerequisite for gaining “insider knowledge” on how to reduce barriers of SDM in mental healthcare. Family members have not often been included in SDM research; however, their experiences and needs are an important part of the decision-making triad. Therefore, we wanted to explore their perspective to identify family-specific needs, including possible differences in perspective from that of affected persons.

The first aim of this qualitative study was to explore how individuals affected by mental illness and their family members experienced decision making in mental healthcare and what their attitudes towards SDM were. We also wanted to know what they needed to successfully participate in SDM: What are the specific barriers and factors facilitating SDM from the perspective of individuals affected by mental illness and their family members? The authors do not limit the term “family members” just to kinship; rather, the broader social network of an individual is encompassed, including all persons with a significant relationship (such as parents, siblings, partners, and close friends), as each of these could provide substantial support in mental healthcare and could, therefore, also be understood as caregivers (following Eassom et al. [33]).

These findings are intended to promote SDM in mental healthcare and to empower affected individuals, as well as their family members, to actively influence their course of treatment in an informed way. Therefore, we want to identify approaches for targeted interventions that could reduce barriers and promote the facilitation of SDM.

## 2. Materials and Methods

### 2.1. Study Design, Participants, Recruitment, and Setting

The present study is part of the research project *Guide2Guide* which, among other aspects, explored experiences with the development and dissemination of guidelines and guideline-based health information, as well as expectations for future guideline development concepts. Within the study, individuals affected by mental illness and family members were also interviewed, as described in Schladitz et al. [34].

This study focuses on the attitudes of adults affected by mental illness and their family members about shared decision making and empowerment in mental healthcare. Participants were interviewed individually via telephone using a semi-structured interview guide (developed by psychological doctoral students and graduates with expertise in qualitative research during a qualitative research workshop). The problem-oriented questioning technique, as developed by Witzel [35], was applied in order to explore subjective perceptions of the topic of decision making. The results are reported according to the Standards for the Presentation of Qualitative Research Results (SRQR) [36].

Respondents were recruited via newsletters of self-help networks for individuals affected by mental illness and family members. Recruitment was supplemented by snow-ball sampling and posters in regional psychiatric practices (see Schladitz et al. [34] for details of the process). Interested parties could contact the study team by e-mail or telephone. The inclusion criteria were as follows: 18 years of age or older, fluent German speaker, affected by mental illness, or related to an individual affected (not restricted by proximity of kinship, and partnership relations were also included). A total of 15 individuals were interested in participating and were sent detailed study information, a consent form, an account data form, and a sociodemographic questionnaire by post in advance. All of them declared their consent in written form and were interviewed.

### 2.2. Data Collection and Analysis

A research assistant who has a degree in psychology and expertise in qualitative research, and a trained and supervised student in the master’s program in psychology conducted *N* = 15 in-depth telephone interviews between August and November 2021. Experiences and attitudes about treatment decisions and empowerment were explored with four initial questions and elaborated in subsequent individual unstructured questions:

*“To what extent do you talk about health information that you researched yourself with your doctor?”*

*“How do you experience the discussion about treatment options?”*

*“In your experience, how are decisions made about upcoming treatments? To what extent does this also apply to treatment decisions regarding your mental illness?”*

*“Where do you see options for improvement in how you can participate in treatment decisions? What would help you in the decision-making process?”*

Interviews were recorded and transcribed verbatim using extended transcription rules for scientific topics. Content was analyzed according to procedures established by Mayring and Fenzl [37] using MAXQDA 2018 by two trained psychological research assistants with experience in qualitative research who independently coded and revised the transcripts. The unit of analysis was either one sentence or a meaningful combination of sentences. The coding scheme was derived deductively from the interview guide categories supplemented by inductive codes derived from topics raised spontaneously and autonomously by the participants. Starting with reading the transcripts multiple times to become familiar with the material, initial codings were assigned and clustered to categories. The researchers reviewed and compared the initial coding and discussed sequences that could not be clearly assigned to codes. Each code was named using content-characteristic words. This process was repeated regularly until a representative coding scheme was generated iteratively. Consensus and methodological rigor were established by mutual agreement between researchers during intermediate steps, as well as in a final group discussion. Data were collected and analyzed in parallel. When no new topics were revealed and the researchers were in agreement, data saturation was achieved, in accordance with procedures established by Glaser and Strauss [38]. When the team assessed that further interviews would not add new information, data collection was stopped after the 15th interview. The interprofessional study team consented to the final coding scheme. For publication purposes, as the interviews were conducted in German, key quotes were translated by an external bilingual professional translation expert and validated by the study team.

### 2.3. Ethical Considerations

The study was granted ethical approval by the Ethics Committee of the Medical Faculty of the University of Leipzig (243/21-ek on 8 June 2021). Participants were informed about the purpose of the study, reporting of study results, and interview recordings. We obtained written informed consent from all participants before data collection. In addition, it was explained that participants could withdraw from the study whenever they wished without any harm. All participants received an allowance of EUR 40 for their time.

Participant were identified with a neutral “project ID” that did not contain any personal information. The interview audio files were stored on a secure drive which was only accessible for the purpose of study evaluation by authorized project members and protected by password. Any personal or geographical references and other information that could allow conclusions about the identity of participants were removed from the transcripts. Data will be deleted after ten years according to the European Union’s (EU) and German General Data Protection Regulation.

## 3. Results

*N* = 4 participants were affected by mental illness themselves, *n* = 5 were family members, and *n* = 6 were affected themselves and additionally had affected family members. Participants were between 30 and 74 years old, most of them (*n* = 12) were female and in most cases affected by affective and anxiety disorders (unipolar depression: *n* = 10, anxiety disorder: *n* = 4; bipolar disorder *n* = 3), and *n* = 2 reported schizophrenia and personality disorders. Sociodemographic data are reported in detail elsewhere [34].

Table 1 provides an overview of the results of the content analyses by presenting categories and (if applicable) subthemes. Questions about SDM and empowerment were associated with a variety of topics: conception of health and mental illness, association between body and psyche, individual implications of mental illness (specifically the process of finding a diagnosis and stigmatization due to mental illness), and experiences and wishes regarding the healthcare system. Specific needs of affected persons and family members regarding the topic complex were explored.

### 3.1. Importance of Understanding the Origins of Mental Illness for SDM

According to some participants, impaired psychological well-being affects a very large part of the population and most people are mentally distressed to varying intensities. Some people are affected to a greater extent and have an insufficient ability to compensate. As a result, these people develop mental symptoms that are considered to be problematic. In these cases, they need help and maybe treatment.

*“Everyone uses some kinds of compensation mechanisms in life. In some people they are more pronounced than in others. Some people have no compensation mechanisms worked out for certain issues, and the situation turns pathological or the burden is too heavy.” *

(B07)

Respondents emphasized that each person must be understood holistically and that there are interactions between body and psyche which are not yet completely understood. When finding a diagnosis and deciding about the adequate treatment, both physical and mental aspects must therefore be considered. Furthermore, severe physical illnesses also impairs mental well-being.

*“A person comes in with severe back problems, so you look at the big picture. […] to take a holistic approach and not just ‘I prod here and it hurts, therefore this is what I’ll be treating.’ You should instead look at the roots […] take time for the patient and then approach the problem holistically: ‘OK, this is what they do for a living. That could well be the cause.’ Even if it’s not obvious, you should also consider the role of the psyche.”*

(B04)

### 3.2. Relevance of Getting a Psychiatric Diagnosis on SDM

Some participants described their concept of a psychiatric diagnosis as a “working hypothesis” and as an attempt to classify existing mental symptoms. Instead, most physicians and therapists communicate a diagnosis as if it were an objective and certain finding. Other participants emphasized that receiving a diagnosis had been helpful and a relieving moment.

*“The formulation back then […] was certainly better than today, because it only mentioned a ‘presence of symptoms’ rather than saying that ‘she has this or that illness.’ That is something I can live with much more easily, and probably other people could too […] But most people, they […] expect a diagnosis so they can say ‘how nice to have a name for it, something to call it.’”*

(B03)

Some participants emphasized that many individuals affected by mental illness perceive a psychiatric diagnosis as a simplification of their symptomatology and, therefore, find it difficult to accept their “label”.

*“The general difficulty with diagnoses is that it’s hard, it’s very vague. I may have symptoms pointing to one illness and symptoms pointing to another. One doctor makes one diagnosis, another doctor makes a different diagnosis, and yet I remain the same person.”*

(B02)

Further, according to the participants, psychiatric diagnoses have practical consequences. These can be negative (e.g., limiting career aspirations—absence of a serious mental illness is a prerequisite for some professions) or positive, as it enables access to resources (e.g., coverage of specific treatments by health insurances).

*“First of all, I find that mental illnesses still get pathologized. It is of course an illness but it also gets stigmatized a little. They won’t give you short-term or permanent disability insurance anymore.”*

(B07)

Some participants wished to focus more on resources and on being involved in the diagnostic process. This involvement should not wait until after the diagnosis has been set and decisions about therapies have been made. This way, patients would better understand of their own mental condition.

*“I filled in a number of questionnaires with my therapist that were about grading or about finding out what areas of life I could still work on or where I might exhibit uncertainty, or what the areas were where I’d done a lot of work. […] I believe that everything that is under your control or that you can do yourself is also an opportunity to learn a lot about yourself.” *

(B04)

Some participants perceived the terms “illness”, “disease”, or “disorder” as problematic, because this reduces people to a label, decreases their confidence and self-perception of their own resources, and impedes their development. Further, mental illnesses are sometimes seen as affecting one’s entire personality. Therefore, therapy and treatment could be perceived as a need to change one’s own person.

*“What actually really annoys me is to keep hearing that one is ill. I was reading something recently that was making quite a bit of sense, but they used words like ‘the ill person’ or ‘the illness’ probably twenty times on every page. People keep zooming in on that without knowing the causes. You can see different doctors and get different diagnoses, even if you describe your situation very clearly. So, you shouldn’t harp on about ‘illness’.”*

(B02)

### 3.3. Importance of Stigma on Being Informed about Mental Illness and SDM

Many participants perceived mental illnesses as stigmatized. They had the impression that a large proportion of society does not regard mental illnesses as a regular element of the disease spectrum, even though they are widespread and not a reason to feel shame. Some also recalled stigmatizing experiences from the past.

*“To have heard it before would have helped me accept and be aware of how I was doing and what it might be that I had. And by that I don’t mean giving it a name, knowing that I have this thing called depression, but rather that it’s something you can get, just like a common cold. I believe it’s up to the educational system to legitimize and improve the understanding of these things, so that you’ve heard about it and you don’t have to feel ashamed of it.”*

(B04)

From the respondents’ point of view, there are differences between various mental illnesses. For example, schizophrenia or psychoses are associated with very negative prejudices, whereas for a disorder like depression, more information is available and therefore more acceptance exists.

*“There is a lot more information available about visible illnesses than there is about invisible ones. And when it comes to the invisible illnesses, there’s too much information on depression and too little on schizophrenia and psychoses. That, in my opinion, is the reason for the terrible notions people have: ‘A person with schizophrenia is likely to kill someone’.” *

(B13)

According to the participants, the stigmatization of mental illnesses also reduces the willingness to seek help if one is affected oneself.

*“Helplines exist, sure, but the hurdle of actually calling one of them is SO high, because I think in these cases many people still feel reluctant to accept help. Or they will feel very weak for seeking help. Or they can’t really get a handle on what’s going on in their minds, because the mind can’t get sick, after all. It’s just something you have to endure.” *

(B04)

Going to a psychiatrist or psychotherapist could be associated with weakness instead of willingness to work through one’s problems.

*“But it has not yet at all been understood that it’s actually good for you to face your illnesses head on. To face your problems head on. I mean, shouldn’t the fact that I’ve been to therapy actually be seen as a sign that I’m prepared to work on my problems.”*

(B07)

Several participants emphasized that information about mental illnesses should be provided as early as possible. If schools would educate students about these topics, there would be less stigma and children and adolescents would know how to seek help.

*“I find it health education in schools VERY important, because there you’ll find the young adults and adolescents. And young people themselves have problems. They have no idea what’s going on with them at that moment or who they can turn to.” *

(B08)

Moreover, according to the participants, many people are not diagnosed with mental illnesses because of stigma. Due to negative associations with mental illnesses, most people know very little about the etiology, associated symptoms, and prevalence of mental disorders. Many people have distorted negative beliefs about them. This may prevent affected individuals from recognizing that their individual symptoms could be a sign of a mental illness. They may not be open to considering the possibility that they are mentally ill themselves and this prevents them from approaching their healthcare provider about diagnosis and help. Information about mental illnesses should, therefore, be provided in all places where people spend time. This way, those affected might recognize their symptoms as a sign of a mental illness and could receive information about help options. It would raise awareness and promote acceptance if those affected by mental illness were able to talk frankly about it.

*“Ever since I’ve been able to deal with it openly, I’ve been extremely open about it with other people, including those who themselves are well, and I’ve talked about these illnesses. I tell people that I have depression and how that presents itself and how hard it is, too. And what happens when you have it, because without information it’s never going to gain acceptance in society. Otherwise you’ll always carry a social stigma, and something absolutely has to be done to fight that.” *

(B01)

### 3.4. Experiencing (Shared) Decision Making

Participants described that they have been involved in treatment decisions with clinicians to varying degrees. Good interpersonal relationships are especially important with psychotherapists; if necessary, one has to search until there is a good interpersonal fit. Once the suitable clinician or therapist was found, however, participants recalled very positive participative experiences.

*“I’m really lucky to have a very good GP, with whom I get along very well on a personal level […], that I can confide in him and that I’ve felt like I am in good hands with him and he’s fortunately told me a great deal. And then later on through my therapist, who knew how to address everything very well and explained many things to me. Before that there was nothing at all.”*

(B04)

Some participants felt it was important to go into a medical consultation with a basic knowledge about one’s own illness in order to be able to discuss needs and preferences, to maintain an overview, and not to relinquish control.

*“I feel it’s important to have a basic understanding even before I see a doctor. So that I can also bring up my own ideas or wishes. Or demands, let’s say. I mean, to know what is possible and what you’re entitled to.”*

(B09)

Some participants emphasized that a decision-making process needs more time than was available in some constellations. Especially in psychiatric hospitals, decisions sometimes have to be made very quickly, without sufficient time to weigh options or to consult with family members.

*“In hospitals, decision-making is very top-down, I mean totally top-down. You may perhaps state your opinion, but often you’re not taken seriously or at least that’s the impression you get. It can also happen that the doctor says: ‘well, there’s really only THIS and THIS and THIS, you are to take THESE drugs.’ What you often don’t have is time to think. […] You have to make your mind up there and then. Most often, there’s no time to sit down and think it through.” *

(B02)

Nevertheless, most participants estimated that the extent to which they can obtain information before making a treatment decision is limited. Without a scientific foundation and corresponding academic education, it is extremely difficult to assess the complex consequences of treatment decisions. Only reading up on something as a layperson is no substitute for academic training and professional experience. The opinion and evaluation of professionals is therefore important and usually is taken into account in one’s own decision-making process.

*“In my experience, professionals build up experience through spending a long time dealing with certain aspects of life to do with health. You cannot always grasp all that in its complexity by reading a set of guidelines or other texts. […] Frankly, I think that there are capabilities acquired through practical experience or training that can’t simply be substituted with ‘oh, I’ll take a couple of weeks and read up on the topic’.”*

(B07)

The participants described a need for comprehensive information and an open dialogue atmosphere when deciding about treatments.

*“Also, just to have the doctor explain why something is done, what is happening and why it’s happening. What is happening in the body? What is the effect, what is supposed to be the effect of a certain therapy? […] Simply to have something explained and cleared up in a one-on-one consultation is helpful. But that doesn’t always happen.”*

(B01)

They also wished to discuss long-term treatment plans and goals, as this would give them a perspective and feeling of security.

*“I always find it very helpful to have a plan like that. It gives you structure and support to know that, okay, ‘if this doesn’t help, there’s also this and there’s that.’ It just gives you a sense of security.” *

(B09)

According to the participants, individualization is important. Recommendations of clinical practice guidelines should be adapted to the individual and his or her specific situation, needs, and preferences. There should be the opportunity and time to weigh the advantages and disadvantages of different treatment options.

*“What I find important, above all, are different perspectives and that everything that can be done, I mean everything that is included in the guidelines and all the different avenues of treatment, that all of that is individually adapted to the patient’s needs. There should be no ‘the patient has to do this or that’, but instead the focus should be on the patient’s interests.”*

(B02)

A trialogical decision-making process involving specialists, individuals affected by mental illness, and family members was considered as optimum.

*“Those are major topics when it’s time to make decisions. Therefore, it has to be all three. It’s best to involve family members, the affected person and specialists.”*

(B02)

### 3.5. Enabling and Hindering Structures to Implement Treatment Decisions

The participants described many good personal experiences regarding involvement in treatment decisions. Further, if other individuals affected by mental illnesses worked as recovery companions or peer supporters in multi-professional teams in clinics, trust would be enhanced. These “experts from experience” could help in the recovery process and would be able to mediate and translate between treatment providers and patients and to strengthen the latter in the process of finding an optimal individual treatment.

*“The information provided by people who are affected is naturally something that, in combination with the information given by specialists, is particularly credible and useful. […] Also, the specialists who work together with people who are affected are often more trustworthy since they tend to have a different attitude. A patient of ours told me that once. She found it great that my boss hired me, as it says a lot about my boss that he would hire recovery companions. I believe she had a point.”*

(B10)

Most participants perceived psychosocial contact and counseling centers as very helpful because appointments were quite quickly available, there was more time to talk than with a physician or therapist, and it felt like being on an equal level. One could use these resources as often as needed. They also recalled very positive experiences with self-help groups; they were knowledgeable, made it possible to evaluate one’s own situation, brought relief, and also opened up important access to information and resources (such as lectures and books).

*“On the other hand, I’ve had positive experiences at places like psychosocial contact and counseling centers because they don’t watch the clock. They really take the time to help you there. The interaction is more at eye level than it is with a doctor or a therapist.”*

(B02)

The participants also reported they had experienced some aspects of the current healthcare system as barriers in implementing treatment decisions. For example, many psychosocial or peer counseling services are only located in urban regions. Participants experience scheduling an appointment with psychotherapists, psychiatrists, and psychiatric clinics as often very difficult, frustrating, and exhausting, and they regard long waiting times for appointments as a significant problem. This presents a major problem, in particular, when drive and concentration are reduced in acute phases of mental illness. In addition, excessive waiting times can lead to the aggravation and progression of symptoms.

*“Following the recommendation to find a therapist or see a specialist, for example. Even just reaching the right people. I mean, it’s nice to know that, under certain conditions, I should see a therapist. But if I can’t make an appointment or I have to go out of my way to see one, it’s an obstacle that I have to face.”*

(B07)

When medical or therapeutic appointments are finally made, they are often unsatisfying because there is often not enough time to talk to the physician or therapist.

*“You always get the feeling there’s not enough time. Everyone has to rush to make a diagnosis. The patient knows too that there are ten other patients waiting outside. When I see my therapist, she has exactly an hour for me and once my time is up, it’s over. I believe time is an important factor.”*

(B06)

The participants also had some ideas for improving the mental healthcare situation. For example, physicians and therapists should spend more time with patients during the decision-making process when it comes to treatment. Treatment providers should explain the causes, symptoms, course of an illness, and treatment options and they should answer questions that arise.

*“On the one hand, I think that if doctors, therapists and the rest of them, the support system, took more time to explain, and also to answer questions.”*

(B06)

According to the participants, psychosocial and self-help initiatives could provide additional and short-term support. Services should be widely known and available also in rural regions. Complementary non-psychotherapeutic options should be expanded for initial relief and first help.

*“I can’t help it that the therapists are all booked up, and I know there’s nothing they can do other than keep on working. I get it. But it’s difficult knowing that you’ve got to wait three or six months for an appointment and not knowing how things are going to go for you in the meantime. And you’re going to be feeling bad in the meantime. So, there should be a place where you can get help at short notice and tips that can actively help you. What can I actually do in this situation? Even if it’s just relaxation techniques.”*

(B06)

### 3.6. Specific Needs of Individuals Affected by Mental Illness and Family Members Concerning SDM

Individuals affected by mental illness expressed that they needed support from family and friends. They do not want to be alone through the process of diagnosis and making and implementing therapy decisions. It could be helpful if family members or friends could accompany them to medical consultations over a longer period of time and help them make informed decisions.

*“Someone from your private life to share the decision with you. […] If a doctor tells me to consider going on medication and I talk about it with a social education worker and we together decide that I ought to give it a try, the problem is that I’m left alone with the decision. […] If you have a family member going through this with you, I think it makes a difference.”*

(B02)

The participants who reported from the perspective of family members did not feel adequately involved in decision-making and therapy processes. They feel that it would make sense to talk to psychotherapists and doctors in clinics by themselves (but there was often not enough time). Further, they would like to sometimes be involved in therapy sessions and to be shown how to provide support. The involvement of family members should be encouraged.

*“Because, in my experience as a family member, I never felt truly involved and I never was really involved. […] I somehow find it absolutely relevant to include the relatives too. I find this approach is missing altogether.”*

(B07)

According to family members, children should also be involved, as they were more aware of the burden on the family than one might think. Excluding children can cause them excess worry and even guilt about the family members illness. Further, children may perceive mental illness as something to be kept secret.

*“My kids are now thirteen and eighteen. They are old enough to put two and two together. They have their points of view and ways of perceiving things. They can say: ‘What’s up with Dad, let’s help him, we’re old enough’.” *

(B14)

However, some family members emphasized that it was also necessary to protect relatives by showing them the limits of what is possible as a caring relative. This can help them from feeling overwhelmed; they can only provide support, and treatment must be provided by professional helpers.

*“I often find myself in a situation where I, as a family member, don’t feel I have the capacity to help. I believe that the problem lies deeper and that the person who is ill and their illness have to be dealt with more intensively. As a family member, I can be open to them, listen to them and so on. Coming up with recommendations is difficult though, maybe because I just don’t have the necessary knowledge. But above all, I’m no psychotherapist and it’s not something I’m able to do. I also don’t think it’s something that the relatives should be doing. […] I think an illness should be treated by professionals.” *

(B07)

## 4. Discussion

### 4.1. Summary

The study results reveal a strong desire for SDM in healthcare and especially in the case of mental illness on the part of those affected and family members, in line with research [28,39,40]. The main barriers to SDM implementation were identified along several dimensions: on the side of the individual affected by mental illness (e.g., information deficits), in communication processes (e.g., between affected individuals and healthcare providers), and structural societal barriers (e.g., stigma of mental illnesses). It became apparent that SDM should not be understood as limited to any one decision.

### 4.2. Early Involvement of Individuals Affected by Mental Illness and Family Members—SDM as an Extended Process

**Figure 1:**
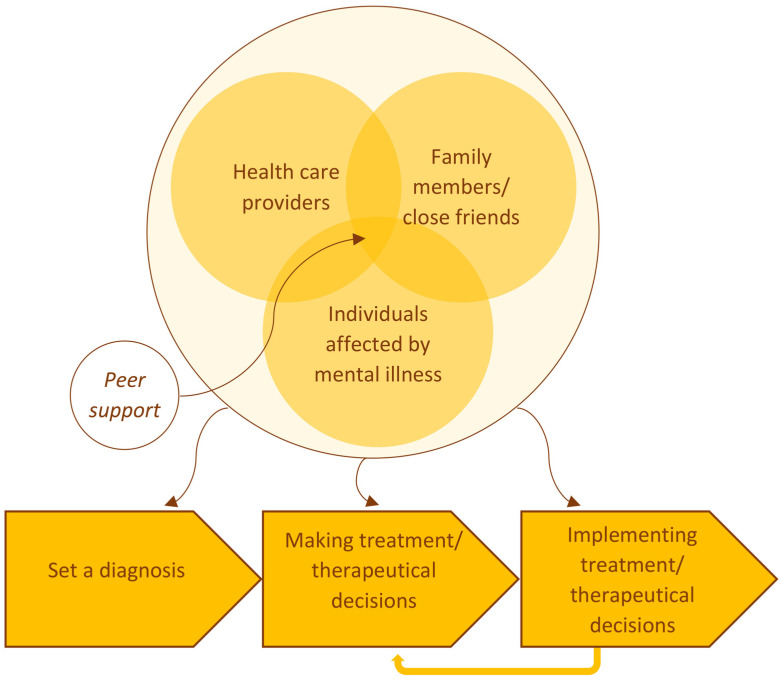
Extended model of shared decision making.

An important new finding of the study is that individuals affected by mental illness and family members should not be involved for the first time *after* diagnosis, when it is time to decide about the treatment; all parties should be involved beginning from the *process of finding a diagnosis.* Extending Gurtner et al. [4], who emphasize the continuous nature of SDM, the shared trialogical approach should therefore include the phases preceding decision making (interpreting symptoms and finding a psychiatric diagnosis) and following the making of a decision (implementing and adjusting or correcting treatment as necessary) (see Figure 1).

The individuals affected by mental illness included in the study perceived the diagnostic process as very complex and difficult and, especially in the case of mental illnesses, as a *simplification of symptoms* and *labeling a person*. Unwanted side effects of clinical diagnoses on self-perception and negative societal reactions have long been described in research literature [41,42]. To decrease negative emotional (e.g., feeling of powerlessness) and behavioral (e.g., reduced help-seeking behavior) consequences, involving affected individuals in interpreting their mental health symptomatology could contribute to a desirable shift in perception of one’s own mental illness, strengthen their recovery orientation, and reveal complementary internal resources, as described by Eads et al. [43].

Likewise, as processes of SDM in the case of mental illnesses include the phase *before* a treatment decision is made, it does not end right *afterwards*. Compliance and adherence is essential in treating mental illnesses and is higher when using an SDM approach [15,44]. Especially for mental illnesses, the decision-making process is extended, e.g., in psychopharmacological therapy, there is an adjustment phase to determine an optimally appropriate drug and dose for the individual [45]. In addition, there is often a multimodal approach (e.g., psychodynamic/cognitive behavioral therapy combined with psychopharmacological therapy) including complementary therapy methods (e.g., art or sports therapy) [46]. Some mental health therapies must continue over a longer period of time, sometimes over a lifetime [45]. The process of SDM in mental illness does not end after a single decision and must also include the phase of implementing a treatment (see Figure 1).

### 4.3. Reducing the Knowledge Gap

The study findings support the view that even though stakeholders would like to practice SDM, impediments such as lack of opportunity and lack of enabling behaviors stand in the way [32]. Being informed about therapy options and the course of treatment is a key pre-requisite for informed decision making according to research [47,48,49]. The existing knowledge gap between healthcare experts (with academic education and experience as a treatment provider) compared to experts from experience (as an individual affected by mental illness or a family member of affected individuals) should therefore be reduced [26,50]. This could be achieved, for example, by providing adapted and evidence-based health information materials, online portals, and decision aids [51,52,53]. However, increased knowledge about relevant health topics alone is not enough; it must be accompanied by interventions which positively influence attitudes about SDM for all stakeholders and aim to balance power in the treatment decision process [32,54]. One approach is aiming interventions at activating and coaching patients before (pre-) healthcare consultations, e.g., collaborative decision skills training [55] or the PREPARE Advance Care Planning Program [56]. But as such interventions are usually quite complex, their implementation in routine healthcare is difficult. There is a need for low-threshold interventions for patients and healthcare providers which should be accompanied by implementation programs.

### 4.4. Barriers of SDM Resulting from the Stigmatization of Mental Illness

As emphasized by the participants and consistent with research, negative societal perceptions of mental illnesses, in particular severe forms, have serious consequences even before a diagnosis is made; many people do not seek information about the symptomatology and the causing and aggravating conditions of mental illnesses [57,58]. As a result, they subsequently have an unrealistic and biased perception of symptoms and treatment options of mental illnesses, e.g., obtained through media information that transports biased conceptions and negative stereotypes of mental illnesses [59]. Because of this lack or incorrect knowledge, an affected individual may not realize that their own symptoms could be a sign of mental illness and thus misinterpret them [60,61]. Additionally, many people are not open-minded to interpreting their symptoms as signs of a mental illness; they simply do not consider that they could be mentally ill because they implicitly fear consequences to their life aspirations [60,62]. As a result, the stigmatization of mental illnesses reduces professional help seeking. In order to avoid a diagnosis, or the “label” and stigma of a mental illness, treatment is not sought [63,64,65]. Negative stereotypes about mental illnesses can also lead to shame and self-stigmatization [62,66] and result in impaired interactions with mental health professionals [67,68].

In accordance with this study, research has shown that stigmatization of mental illnesses is a major barrier for SDM [39,67,68]. Educational strategies, media guidelines, family engagement campaigns and health information materials may be able to achieve even more with mental illnesses than with non-stigmatized physical illnesses by providing evidence-based information. Participants emphasized that the positive effect of health information on empowerment not only had an impact on those individuals affected by mental illness, but also on the society as a whole; scientifically based and easily understandable health information had an educational effect and consequently could reduce existing prejudices and biased perceptions about mental illnesses, as well as increase mental health literacy [60,69,70,71]. Approaches that include positive personal contact are more efficient than educational and media-based approaches without direct contact [72,73,74,75].

### 4.5. Structural Barriers of SDM

Another comprehensive class of barriers to SDM relates to the healthcare system itself, e.g., the insufficient number of available medical mental health specialists and long waiting times for a medical appointment. Although many of these mentioned barriers can only be addressed at the structural level of the care system with more fundamental changes, there are, nevertheless, interventions that could be adapted rather quickly and within the existing structures.

Since trustful communication with physicians and therapists was mentioned as a key prerequisite for SDM by participants (in accordance with Aoki [39]), but lack of time during a medical or therapeutical consultation was often reported as impeding [25,76], information materials and decision-making aids could help clinicians in the task of sharing information with individuals affected by mental illness and family members and therefore support SDM [77]. These materials could save time during the consultation; when a patient or client arrives informed, he/she can ask specific questions. Perhaps the patient could discuss treatment options with family members in advance and could clarify his/her own needs and preferences [39]. Digital technology can provide promising options for individual adaptation, enhancement, and dissemination of health-related information and decision aid materials for supporting SDM [78,79]. There are findings that digital SMD interventions had positive effects, e.g., on patient activation, symptomatology, the working alliance with treatment providers, and the decision process [80].

Spatz et al. [49] also emphasize that trustful communication with a clinician is necessary, but not sufficient to fully convey information for informed decision making, which requires supplementary written information and decision aids for laypersons. Adequate adaption is necessary, involving individuals affected by mental illnesses and taking into account their specific needs (which were described in Schladitz et al. [34]). Innovative approaches such as open notes in electronic health records also have the potential to improve trustful communication with clinicians and to increase empowerment and treatment adherence [81].

### 4.6. Peer Support and Self-Help-Promoting SDM and Complementary Support Offers

An important finding of our study is that peer support is highly relevant. These “experts from experience” [82] and self-help associations were described as important sources of valuable and specific information that could alleviate these barriers to some extent, and as an important element of empowerment and strengthening the recovery perspective [46,83]. These structures can mediate between clinicians and individuals affected by mental illness and their family members, and they can increase trust in healthcare providers [82,84,85,86] (see Figure 1). Peer support, self-help associations, and also psychosocial counseling services (which were also rated very positively) should therefore be further strengthened within the healthcare system, advertised in information campaigns, and prominently referred to within health information [87]. Peer support also has a positive impact on personal-level barriers (as described above) of self-stigma and stress caused by stigmatization [88].

Participants also emphasized the importance of becoming active on their own, to “do something” while waiting for clinical or therapeutical appointments. Complementary healthcare offers could provide first help and relief and should therefore be made accessible. E-mental health programs—which could also be used by those who are unable to attend therapeutic appointments due to rural residence, mobility limitations, or lack of time—could help in reaching underserved groups [89,90,91].

### 4.7. Strength and Limitations

In-depth interviews were chosen as the method of data collection because they provided the opportunity to explore different perspectives on health information in the context of SDM. Individuals affected by mental illness, family members, and people taking both perspectives could talk freely about their unique experiences, expectations, and needs regarding the topic of SDM in mental healthcare. By using a semi-structured interview guide, participants had the opportunity to raise new topics which have not been previously specified by the researchers.

In qualitative research, the external validity of a study including transferability and representativeness of the results must be critically reviewed. Therefore, the size and composition of the sample should be adequate and sufficiently varied [92]. The generalization of the findings requires caution as this study was conducted in Germany and the experiences and attitudes may be specific to the national mental healthcare situation. As the majority of participants reported affective and anxiety disorders in themselves or their family members, the perspective of people with psychiatric diagnoses such as obsessive–compulsive disorders, addictive disorders, etc., and their family members and friends could not be explored. Furthermore, the study did not include any immigrants, non-Germans, adolescents, or adults younger than 30 years old. People with lower educational backgrounds and males are underrepresented. The challenging recruiting process (as described in Schladitz et al. [34]) could be another possible source of sample bias. The study population of individuals affected by mental illness and family should be regarded as a hard-to-reach population; therefore, recruitment is complex and a variety of specific and approved measures must be considered for this special population [93]. An additional study could specifically focus on people with other mental illnesses, e.g., psychosis, obsessive–compulsive, and addictive disorders.

However, the sample was reasonably balanced with approximately the same number of individuals affected by mental illness, family members of affected individuals, and people for which both applied. The sample size was appropriate [94,95] and the interviews produced saturated data. To enhance the credibility, the authors used a systematic procedure and discussed the coding process and categories. Quotations were included in the results section in order to increase the confirmability of the study [37,96].

## 5. Conclusions

This exploratory study provides some insights into the experiences and expectations of individuals affected by mental illness and their family members, as well as the needs, barriers, and facilitators of SDM.

- In order to promote SDM, the knowledge gap between healthcare providers and individuals affected by mental illness and relatives should be reduced, e.g., by providing target group-tailored information and decision aids.
- Interventions should be developed and implemented which positively influence attitudes toward SDM in affected individuals, family members, and healthcare providers and which balance power in the treatment decision process, e.g., by low-level pre-consultation interventions.
- As stigmatization of mental illnesses is a significant barrier to SDM, destigmatization should be increased by implementing educational strategies and family engagement campaigns and disseminating media guidelines and evidence-based health information.
- Peer support, self-help associations, and psychosocial counseling services can promote empowerment and strengthen the recovery orientation. They should, therefore, be further strengthened within the healthcare system, advertised in information campaigns, and prominently referred to within health information.
- Complementary healthcare offers and e-mental health programs have the potential to empower and to spur action for recovery. Therefore, they should be made more accessible.

This study indicates that individuals affected by mental illness and their family members have a strong desire for SDM. Barriers are potentially modifiable and a variety of approaches for interventions in the psychiatric sector could be identified. This study also shows the importance of understanding SDM as a process that should begin at the diagnostic phase.

## Figures and Tables

**Table 1:** Overview of the categories and subthemes.

Category	Subtheme
1 Importance of understanding the origins of mental illness for SDM	
2 Relevance of getting a psychiatric diagnosis on SDM	
3 Importance of stigma on informedness about mental illness and SDM	3.1 Stigmatization
	3.2 Destigmatization
4 Experiencing (shared) decision making	4.1 Past experiences
	4.2 Ideas for improving the decision-making process
5 Enabling and hindering structures to implement treatment decisions	5.1 Good experiences
	5.2 Criticism
	5.3 Ideas for improving
6 Specific needs of individuals affected by mental illness and family members concerning SDM	6.1 Specific needs of affected persons
	6.2 Specific needs of family members

## Data Availability

All data generated or analyzed during this study are available from the corresponding author on request.
